# Interrogating the superconductor Ca_10_(Pt_4_As_8_)(Fe_2−x_Pt_x_As_2_)_5_ Layer-by-layer

**DOI:** 10.1038/srep35365

**Published:** 2016-10-14

**Authors:** Jisun Kim, Hyoungdo Nam, Guorong Li, A. B. Karki, Zhen Wang, Yimei Zhu, Chih-Kang Shih, Jiandi Zhang, Rongying Jin, E. W. Plummer

**Affiliations:** 1 Department of Physics and Astronomy, Louisiana State University, Baton Rouge, LA 70803, USA; 2 Department of Physics, The University of Texas, Austin, TX 78712, USA; 3 Brookhaven National Laboratory, Upton, NY 11973, USA.

## Abstract

Ever since the discovery of high-T_c_ superconductivity in layered cuprates, the roles that individual layers play have been debated, due to difficulty in layer-by-layer characterization. While there is similar challenge in many Fe-based layered superconductors, the newly-discovered Ca_10_(Pt_4_As_8_)(Fe_2_As_2_)_5_ provides opportunities to explore superconductivity layer by layer, because it contains both superconducting building blocks (Fe_2_As_2_ layers) and intermediate Pt_4_As_8_ layers. Cleaving a single crystal under ultra-high vacuum results in multiple terminations: an ordered Pt_4_As_8_ layer, two reconstructed Ca layers on the top of a Pt_4_As_8_ layer, and disordered Ca layer on the top of Fe_2_As_2_ layer. The electronic properties of individual layers are studied using scanning tunneling microscopy/spectroscopy (STM/S), which reveals different spectra for each surface. Remarkably superconducting coherence peaks are seen only on the ordered Ca/Pt_4_As_8_ layer. Our results indicate that an ordered structure with proper charge balance is required in order to preserve superconductivity.

Since the discovery of Fe-based superconductors in 2008, tremendous effort has been expended to understand the origin of their physical properties, that exhibit strong coupling between structure, magnetism, and superconductivity. Similar to high-temperature (T_c_) cuprate superconductors, Fe pnictide superconductors form a layered structure with Fe_2_As_2_ building blocks. Depending on the spacers that separate these building blocks, these superconductors are often categorized as “111” (e.g. LiFeAs)^1^, “1111” (e.g. LaFeAsO and CaFeAsF)^2^,^3^, and “122” (e.g. BaFe_2_As_2_)^4^ families etc. Surprisingly, these compounds exhibit only small anisotropy in their physical properties, in spite of their layered structure^5^,^6^,^7^,^8^,^9^. This suggests strong Fe_2_As_2_ interlayer coupling. Cleaving a single crystal of such a system usually results in the Fe_2_As_2_ layer covered by atoms from the spacer, thus difficult to study the role a spacer plays using surface sensitive techniques. Recently, a new Fe-based superconducting system Ca_10_Pt_n_As_8_(Fe_2_As_2_)_5_ with n = 3 (Ca10-3-8) and n = 4 (Ca10-4-8) has been reported^10^,^11^,^12^. As shown in Fig. 1a there is additional Pt_n_As_8_ layer sandwiched in between two Ca layers within adjacent Fe_2_As_2_ building blocks: Ca10-3-8 is triclinic, while three different structures—tetragonal, triclinic, and monoclinic—have been reported for the Ca10-4-8 compound^10^,^11^,^12^,^13^. According to first-principles calculations^11^,^14^,^15^ and an angle-resolved photoemission spectroscopy study^16^, these Pt_n_As_8_ layers are conducting, in contrast to insulating spacers in 111, 1111, 122 families, and more complex compounds such as Sr_3_Sc_2_O_5_Fe_2_As_2_^17^ and Sr_4_V_2_O_6_Fe_2_As_2_^18^. Thus, this new Ca_10_Pt_n_As_8_(Fe_2_As_2_)_5_ system offers an excellent platform to explore the role of interlayer spacers in interlayer superconductivity.

In this paper, we report experimental investigations of superconducting Ca_10_Pt_4_As_8_(Fe_2−x_Pt_x_As_2_)_5_ single crystals layer-by-layer using scanning tunneling microscopy and spectroscopy (STM/S). The layer-by-layer probing of these layered superconductors is extremely important as illustrated in the recent study of Bi_2_Sr_2_CaCu_2_O_8+δ_, where it was found that “the well-known pseudogap feature observed by STM is inherently property of the BiO planes and thus irrelevant directly to Cooper pairing”^19^. By creating a fresh surface through single-crystal cleavage under ultra-high vacuum, one can study both structural (STM topography) and electronic properties (scanning tunneling spectroscopy (STS)) of the exposed surfaces. By comparing STS spectra taken on different layers, we address how the surface structure and Ca concentration affect superconductivity on the surfaces of Ca10-4-8.

## Results

**Figure 1:**
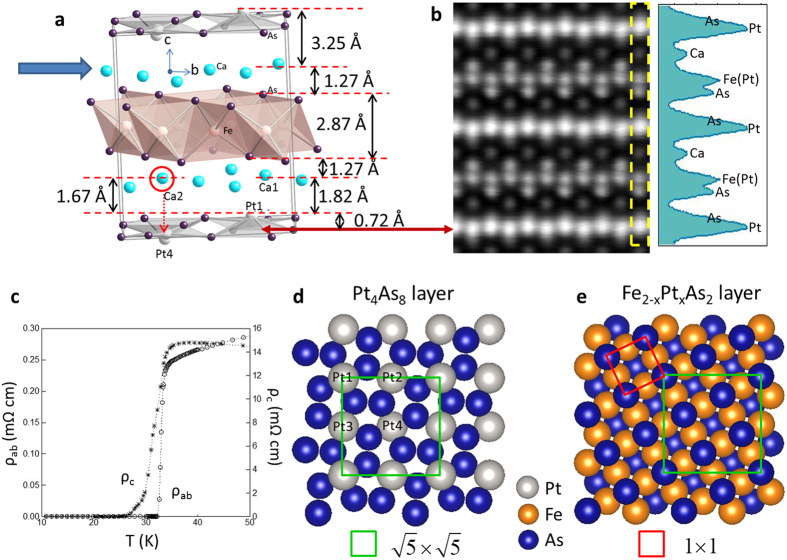
Structure of the Ca10-4-8. (**a**) Schematic of the Ca10-4-8 bulk structure with the relative spacings between the different planes. Preferred cleavage plane is marked with a blue arrow. (**b**) HAADF-STEM image taken along [210] direction shows detailed structure of Ca_10_Pt_4_As_8_(Fe_2−x_Pt_x_As_2_)_5_: one of Pt_4_As_8_ layers and the corresponding plane in (**a**) are marked with a red arrow. Intensity profile measured from the yellow rectangle provides the configuration of atomic planes stacked along c direction: alternating Pt doped Fe_2_As_2_ and Pt_4_As_8_ layers with Ca ions in between them. Double stack of the structure in Fig. 1a is shown in the image. (**c**) In-plane resistivity (*ρ*_*ab*_) shows a clear superconducting transition with onset T_c_ = 34 K and zero resistivity T_c_ = 31 K. Out-of-plane resistivity (*ρ*_*c*_) has slightly broad superconducting transition with a peak near 38 K. Bulk-truncated Pt_4_As_8_ layer (**d**) and Fe_2−x_Pt_x_As_2_ layer (**e**). The unit cell of each surface is marked with green (Pt1-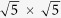) and red (1 × 1) squares.

For the Ca_10_Pt_4_As_8_(Fe_2−x_Pt_x_As_2_)_5_ single crystals we used for this study, an atomically-defined layered structure is clearly imaged by high-angle annular dark field scanning transmission electron microscopy (HAADF-STEM) taken along [210] direction (Fig. 1b). Similar to previous report^12^, our crystals form a tetragonal structure with a lattice parameter *a* = 8.733 Å, and interlayer distances indicated in Fig. 1a. The intensity of HAADF-STEM images strongly depends on the averaged atomic number (Z) in the projected atomic columns. As shown in Fig. 1b, the higher intensity of Fe (Z = 26) column than that of As (Z = 33) column indicates that there exists some Pt in Fe_2−x_Pt_x_As_2_ layer, i.e. *x* ≠ 0. Noticeable Pt doping in Fe_2−x_Pt_x_As_2_ layers was reported in previous studies^10^,^12^, which is responsible for various reported T_c_ values of the material. For our study, Ca_10_Pt_4_As_8_(Fe_2−x_Pt_x_As_2_)_5_ single crystals exhibit a superconducting transition at T_c_ = 34 K, as shown in both the in-plane and out-of-plane resistivity in Fig. 1c.

Fresh surfaces were created by cleaving single crystals under ultrahigh vacuum (~10^−10^ torr) at ~90–100 K. Since Ca atoms are weakly bounded to adjacent layers but closer to the Fe_2−x_Pt_x_As_2_ layer than the Pt_4_As_8_ layer (see Fig. 1a), naively two surface terminations should be expected with roughly the same probability: (1) a full Ca layer on the top of Fe_2−x_Pt_x_As_2_ and (2) a bare Pt_4_As_8_ layer. Bulk-truncated Pt_4_As_8_ layer and Fe_2−x_Pt_x_As_2_ layer with their relative surface unit cells are shown in Fig. 1d,e, respectively. The Fe_2−x_Pt_x_As_2_ surface has a (1 × 1) structure with 
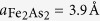
. Since the *x* value is yet to be determined, showing Fig. 1e is the structure of Fe_2_As_2_ without indicating the location of Pt. (marked with a red “square” (□) represents a (1 × 1) structure in Fig. 1e). The Pt_4_As_8_ surface has a different (1 × 1) structure with 
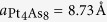
 (marked with a green “square” (□)), which has the same size as the primitive unit cell for bulk-truncation (Fig. 1a). This green “square” crystal unit cell for Pt_4_As_8_ is commensurate with the surface (1 × 1) Fe_2−x_Pt_x_As_2_ lattice. To facilitate consistent notation every structure will be denoted relative to the (1 × 1) Fe_2−x_Pt_x_As_2_ surface unit cell: the bulk-truncated (1 × 1) Pt_4_As_8_ structure (Fig. 1d) will be denoted as the Pt1-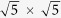 structure. All observed structures are summarized in Table 1, which will be presented and discussed later.

**Figure 2:**
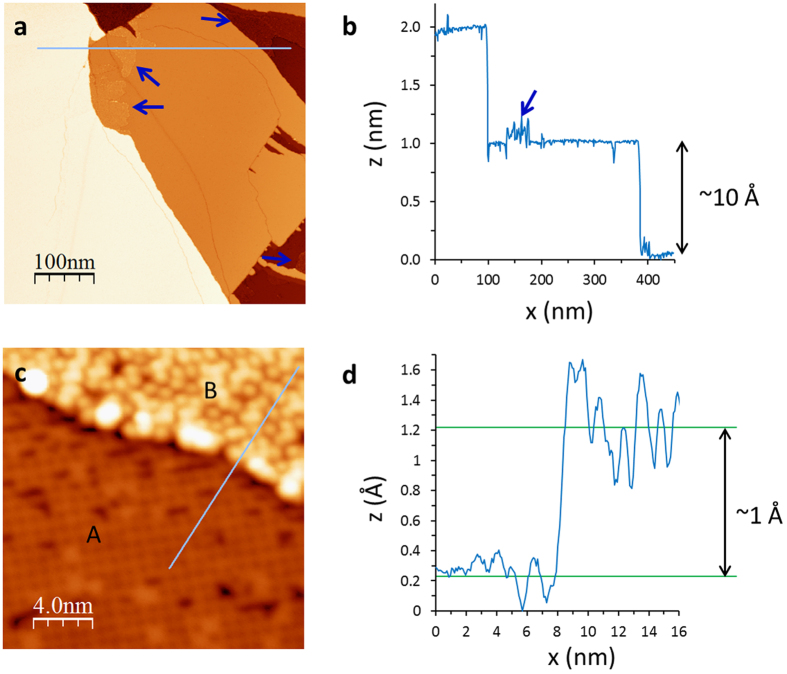
Pt1-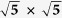 surface and Ca2-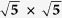 surface. (**a**,**b**) STM image (taken at 4.3 K) of the stepped Pt_4_As_8_ surfaces (V_sample_ = 0.7 V, I = 2.3 pA) and a line profile showing steps with a reported unit cell height^11^,^12^. Ca covered areas are marked with arrows. One of such Ca covered area is shown in (**c**): STM image (taken at 4.3 K) showing both Pt1-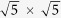 surface (the region A) and Ca2-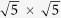 surface (the region B) (V_sample_ = 0.2 V, I = 2 pA). (**d**) Line profile shows Pt1-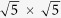 layer with a small step height (~1 Å) to the Ca2-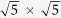 surface.

**Figure 3:**
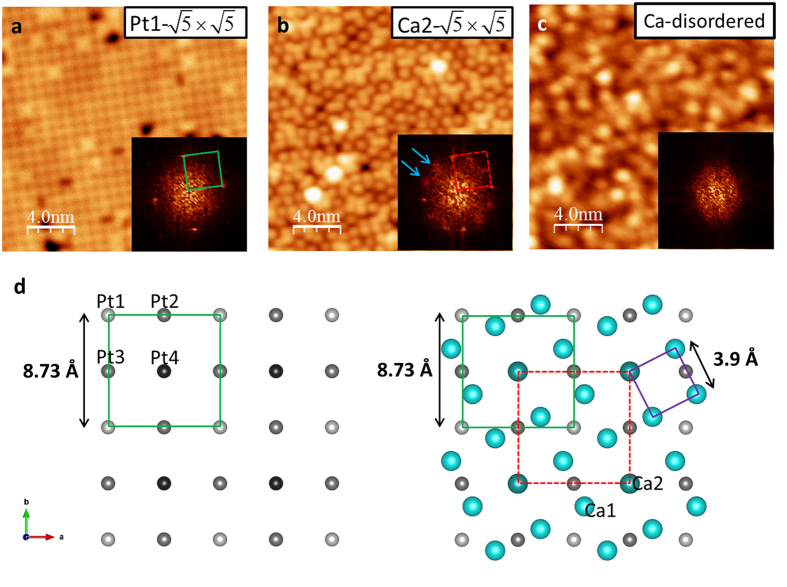
Observed surfaces and their relative structures. STM image of (**a**) Pt1-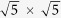 surface (V_sample_ = 0.7 V, I = 2.4 pA), (**b**) Ca2-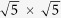 surface (V_sample_ = 0.2 V, I = 2 pA), and (**c**) disordered Ca covered Fe_2−x_Pt_x_As_2_ layer (V_sample_ = 0.2 V, I = 2.4 pA). All images are taken at 4.3 K. (insets) corresponding Fourier transform patterns. (**d**) Schematic shows relative relations of each surface structure. The green solid square represents 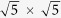 unit cell of the Pt1-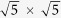 surface (as also shown in Fig. 1d), the purple solid square represents (1 × 1) structure of Ca layer with a full-monolayer (ML) coverage, and the red dashed square is 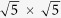 unit cell of the Ca2-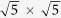 surface. Pt1 (Pt4) is located at 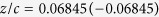 above (below) the Pt in-plane (Pt2, Pt3) at 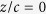. Ca2 atoms forming 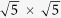 unit cell are located at 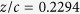, which is ~0.15 Å lower than other Ca atoms (Ca1 at 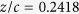).

Figure 2a shows the STM image of cleaved surfaces, displaying large flat terraces. Figure 2b shows the line profile recorded along the marked location in Fig. 2a, where the step height is ~10 Å, which corresponds to a reported unit cell height^11^,^12^. In addition to these large steps, there are regions that show much smaller steps (~1 Å). These are marked with arrows in Fig. 2a,b. The magnified image of such an area is shown in Fig. 2c. Notably, it consists of two terraces (A and B) with a height difference of only ~1 Å (see Fig. 2d). The observed surfaces can be identified using structural information displayed in Fig. 1a. Figure 3a shows the topography of region A marked in Fig. 2c. Note it has a “square” unit cell with a lattice size ~8.7 Å with some local electronic inhomogeneity (Supplementary Fig. S1). This is identical to the lattice size of the bulk-truncated (1 × 1) Pt_4_As_8_ structure (Fig. 1d, “Pt1-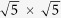
”). Thus, we conclude that the region A is the Pt_4_As_8_ layer without reconstruction nor the presence of Ca. As shown in Fig. 1a,d, there are four Pt sites in the crystal unit cell: Pt1 is above the plane formed by Pt2 and Pt3, and Pt4 is below the plane. STM should observe primarily the Pt1 atoms, forming the “square” unit cell with the edge length of ~8.7 Å, as illustrated in Fig. 1d (green square) and labeled Pt1-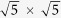.

Since the step height between regions A (Pt_4_As_8_) and B is ~1 Å, the region B can only be a Ca covered area on the top of Pt_4_As_8_. According to Fig. 1a, there are five Ca atoms on one Pt1-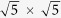 unit cell: four (Ca1) are located at *z/c* = 0.2418 and one (Ca2) at *z/c* = 0.2294 (where *z/c* = 0 is the plane formed by Pt2 and Pt3)^12^. As illustrated in Fig. 3d, the weakly bounded Ca layer with a full-monolayer (ML) coverage should have a (1 × 1) structure with the lattice parameter of 3.9 Å (where 1 ML is defined as the amount of Ca atoms: four Ca1 and one Ca2 per primitive unit cell), the same structure as the bulk-truncated (1 × 1) Fe_2−x_Pt_x_As_2_ (Fig. 1e). However, the region B (see Fig. 3b) shows a different structure than the one with a full ML coverage. The Fourier transform pattern in the inset of Fig. 3b shows the same spots as observed in Pt1-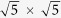 (see the inset of Fig. 3a): Ca2 atoms form a “square” unit cell with the lattice size of ~8.7 Å, which is commensurate with the Pt1-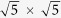 underneath. While we expect to observe a fully Ca covered Fe_2−x_Pt_x_As_2_ layer and a bare Pt_4_As_8_ layer, the existence of Ca2-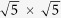 can be traced back to the Ca distribution along the *c* axis. Ca2 is ~0.15 Å closer to the Pt_4_As_8_ surface compared to Ca1, and is located on the top of Pt4 (Figs 1a and 3d). When the crystal is cleaved, Ca2 atoms remain on the Pt_4_As_8_ surface while Ca1 atoms are peeled off. The resultant Ca2-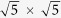 unit cell is marked in Fig. 3d with the red dashed square. By counting atoms shown in Fig. 3b, the Ca coverage is estimated to be ~0.17 ML, which is close to 0.2 ML, the amount of Ca2 to form a perfect 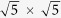 structure (Ca1:Ca2 = 4:1). However, the additional weak and broad spots marked with arrows in the Fourier transform patterns (see the inset of Fig. 3b) indicate that there is additional short-range order formed by Ca atoms.

In addition to the bare Pt1-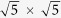 (region A) and Ca2-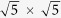 (regions B) surfaces, we observe another surface. As shown in Fig. 3c, this surface does not form any ordered structure. As will be discussed later, this is identified as a Ca covered Fe_2−x_Pt_x_As_2_ layer (“Ca-disordered”).

**Figure 4:**
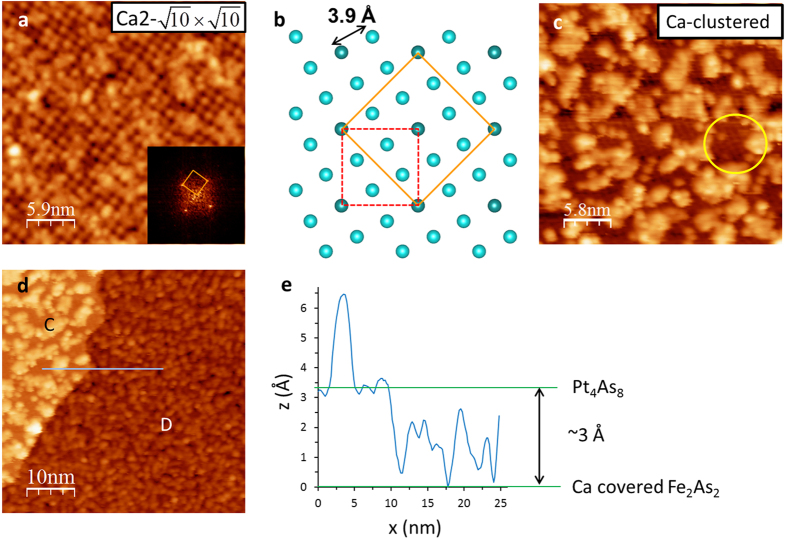
Annealing effect on Ca2-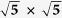 and disordered Ca surfaces. (**a**,**b**) STM image (taken at 4.3 K) of Ca2-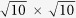 surface after warmed from 4.3 K to room temperature (~290 K) and schematic of the structure (V_sample_ = 100 mV, I = 2 pA). (inset) Fourier transform pattern. STM image of Ca2-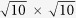 surface taken at 80 K is shown in Supplementary Fig. S3: there is no significant difference between images taken at 4.3 K and 80 K. (**c**) Additional hour long annealing at room temperature causes surface Ca atoms become clustered, revealing underneath Pt1-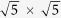 surface (taken at 80 K, V_sample_ = 1 V, and I = 100 pA). One of such revealed Pt1-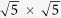 surfaces is marked with a yellow circle. (**d**) STM image showing two different layers (taken at 80 K, V_sample_ = 1 V, and I = 100 pA): Pt1-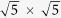 with Ca clusters (the region C) and disordered Ca surface on Fe_2−x_Pt_x_As_2_ layer (the region D). (**e**) Distance between Pt_4_As_8_ layer and Ca covered Fe_2−x_Pt_x_As_2_ layer is shown in the line profile.

Returning to the Ca2-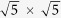 surface, we now illustrate that it is metastable. In the scanned area of ~9 μm^2^ from 3 different as-cleaved samples, Pt1-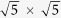 surface (86%) and Ca2-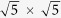 layer (14%) on top of Pt1-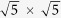 surface are observed. In some Ca2-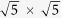 regions, Fourier transform patterns show stronger additional spots (marked with arrows in the inset of Fig. 3b) with weaker 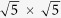 spots, indicating the Ca2-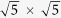 surface is not a ground state. Figure 4a shows the Ca layer after the sample is warmed from 4.3 K to room temperature (~290 K) for an hour, revealing a new structure with a 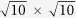 unit cell (“Ca2-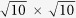
”) displayed in Fig. 4a,b. Ca2-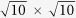 is the (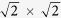)R45° reconstruction of the original Ca2-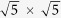 unit cell, suggesting the Ca surface undergoes a structure change during the warming process. Since the Ca coverage for the perfect Ca2-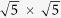 structure is 0.2 ML, there are excess Ca atoms (0.1 ML) after forming Ca2-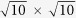 structure. These excessive Ca atoms form clusters on the surface as shown in Fig. 4a. With an additional hour-long annealing at room temperature, all surface Ca atoms are clustered (“Ca-clustered”), revealing the Pt1-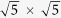 surface underneath (Fig. 4c and the region C in Fig. 4d): the square lattice of Pt1-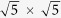 surface can be seen in between clusters (one of such area is marked with a yellow circle in Fig. 4c). Thus, the Ca-clustered surface is stable and the Ca2-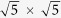 and Ca2-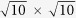 surfaces are metastable.

As for the Ca-disordered Fe_2−x_Pt_x_As_2_ surface (Fig. 3c), it remains disordered after annealing (Fig. 4d). According to the line profile shown in Fig. 4e, the step height between regions C and D is ~3 Å. Since the region C is the Pt_4_As_8_ layer, the region D must be the Ca layer on the top of Fe_2−x_Pt_x_As_2_ layer. We also observed the same disordered surface located about 3.5 Å lower than the Ca2-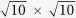 surface (Supplementary Fig. S2), after initial warming up from 4.3 K to 290 K. The morphological feature is also the same as that shown in Fig. 3c, which is obtained from the as-cleaved Ca/Fe_2−x_Pt_x_As_2_ surface. For comparison, the surface of CaFe_2_As_2_ has a (1 × 2) structure formed by 0.5 ML Ca on the top of Fe_2_As_2_ layer^20^. For Ca_10_Pt_4_As_8_(Fe_2−x_Pt_x_As_2_)_5_, the absence of ordered Ca/Fe_2−x_Pt_x_As_2_ surface is undoubtedly due to the asymmetric interlayer spacing: the Ca layer is closer to the Fe_2−x_Pt_x_As_2_ layer than to the Pt_4_As_8_ layer. When the sample is cleaved, most of Ca atoms stay on the top of Fe_2−x_Pt_x_As_2_ layer, which are disordered as shown in Fig. 3c (as-cleaved) and 4d (after annealing). In contrast to the Ca/Pt_4_As_8_ surface, the disordered Ca/Fe_2−x_Pt_x_As_2_ surface is insensitive to annealing. Only the surface corrugation changes from ~1 Å (as-cleaved) to ~2 Å (after annealing). The large amount of disordered Ca atoms prevents from seeing the bare Fe_2−x_Pt_x_As_2_ layer.

Cleaving a Ca_10_Pt_4_As_8_(Fe_2−x_Pt_x_As_2_)_5_ single crystal results in three surfaces and two additional surfaces via annealing as summarized in Table 1. In the Ca2-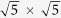 surface, the step height (~1 Å) observed in Fig. 2d is ~0.7 Å shorter than the corresponding bulk spacing between Ca2 and Pt1 (1.67 Å). This STM result is consistent with the behavior reported at the surface of CaFe_2_As_2_ with 0.5 ML Ca coverage^20^, where Ca atoms are pulled down by ~0.5 Å (~30%) determined by low energy electron diffraction (LEED). In addition, the calculated charge states using the Bader scheme in ref. 15 [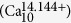, 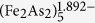, and 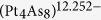] can be utilized to support the large inward relaxation. In bulk, positively charged Ca atoms are positioned in between two negatively charged layers, Fe_2−x_Pt_x_As_2_ and Pt_4_As_8_. When the negatively charged Fe_2−x_Pt_x_As_2_ layer is removed by cleaving there would be too many positive charged Ca atoms unless some are removed with the Fe_2−x_Pt_x_As_2_ layer. If the remaining Ca is indeed above Pt4 atom, this specific Ca atom (Ca2) has more room to be pulled down than other four Ca atoms (Ca1) since Pt4 is located at 0.72 Å below the plane formed by Pt2 and Pt3 at *z*/*c* = 0. In the Ca-disordered surface, the remaining Ca atoms do not form any ordered structure as shown in Figs 3c and 4d. This may be the reason that the observed spacing (~3 Å) between Pt_4_As_8_ layer and Ca covered Fe_2−x_Pt_x_As_2_ layer (Fig. 4e) is close to the spacing in the bulk (3.25 Å).

**Figure 5:**
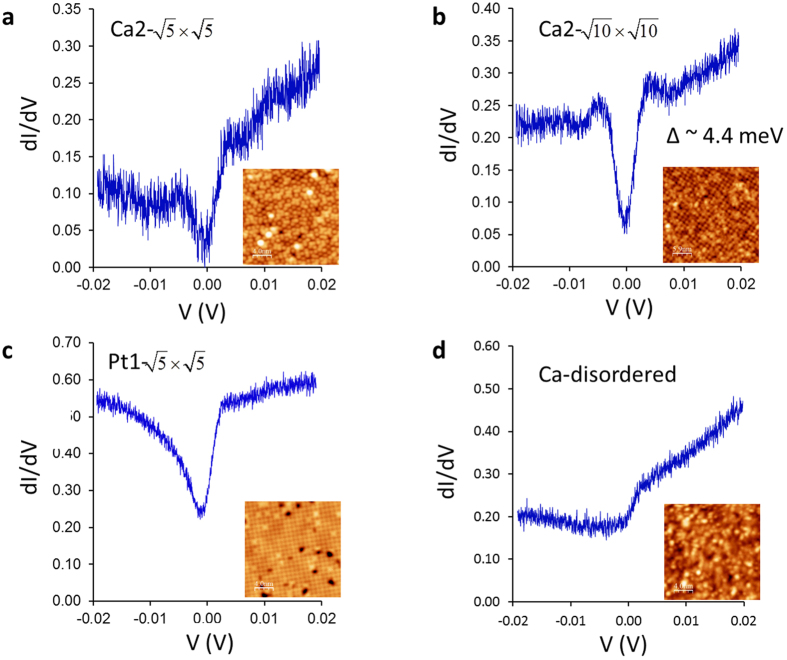
STM images of four observed surfaces (inset) and spectra corresponding to each surface taken at 4.3 K: (a) Ca2-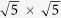, (b) Ca2-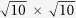, (c) Pt1-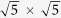, and (d) Ca-disordered. Only on the ordered Ca surfaces, superconducting features are observed. Note that spectra are raw data and the tip was stabilized at the sample bias V_sample_ = 20 mV and the tunneling set current (**a**) 15 pA, (**b**) 20 pA, (**c**) 40 pA, and (**d**) 25 pA, respectively.

The most significant observation on the cleaved Ca10-4-8 surfaces comes from the STS measurements. From the bulk measurements, T_c_ of Ca10-4-8 is ~34 K (see Fig. 1c). Thus, STS measurements at a much lower temperature (4.3 K) than T_c_ are expected to show the opening of superconducting energy gap. This is indeed observed on the ordered Ca surfaces on Pt_4_As_8_. Figure 5a,b show spectra taken on the Ca2-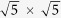 and Ca2-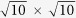 surfaces with corresponding STM images (insets). The annealed surface (Ca2-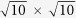) shows enhanced superconducting coherence peaks (Fig. 5b) when compared to the spectrum taken on the as-cleaved surface (Ca2-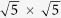) (Fig. 5a). Note that the spectra shown in Fig. 5 are raw data (no normalization) so information about absolute conductance cannot be obtained. However, from the coherence peak positions in Fig. 5b, one can estimate the energy gap Δ ~4.4 meV at T = 4.3 K. This results in that 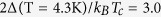. According to the BCS theory, 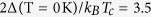 for weakly coupled superconductors. The smaller 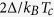 may result from underestimated Δ due to finite temperature and/or overestimated T_c_ at the surface. It is worth pointing out that the spectrum (Fig. 5b) exhibits a finite zero bias conductance (ZBC). Such a feature has been observed previously in other Fe-based superconductors^20^,^21^,^22^,^23^,^24^,^25^,^26^, even when probed at much lower temperatures than their T_c_, indicating the ZBC is not due to the thermal broadening effect. It was also shown that spectral features can vary on different reconstructed surfaces in BaFe_2−x_Co_x_As_2_^26^ suggesting reconstructions may be closely related to the observed ZBC in Fe-based superconductors. The origin of the ZBC, however, is not clear at the moment and requires further investigation.

The STS data for the Pt1-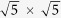 surface shows a kink with positive bias but smooth *dI*/*dV* with negative bias (Fig. 5c). In addition, the detailed feature in each spectrum varies significantly depending on the site where STS is taken (Fig. S4). Likewise, the STS spectra taken on the Ca-disordered (on the Fe_2−x_Pt_x_As_2_) surface lack any features that could be associated with superconductivity (Fig. 5d). These spectra are strongly site dependent as well.

## Discussion

What preserves superconductivity on the Ca-ordered Pt_4_As_8_ surfaces (Ca2-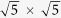 and Ca2-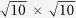) but diminishes superconductivity on the bare Pt_4_As_8_ surface (Pt1-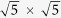) and Ca-disordered Fe_2−x_Pt_x_As_2_ surface? It is well known that the Fe_2_As_2_ layer is responsible for superconductivity in bulk Fe-based superconductors, and the Pt_4_As_8_ layer is expected to be superconducting as well, given the nature of 3D superconductivity (Fig. 1c). We recall that superconducting coherence peaks have been observed in spectra taken on the surfaces of other Fe-based superconductors^20^,^23^,^27^, in which the surfaces are ordered. For example, the cleaved surface of Ca(Fe_0.925_Co_0.075_)_2_As_2_ has an ordered stripe phase formed by a half monolayer Ca on the Fe_2_As_2_ layer^20^. Moreover, superconducting features are enhanced in the Ca2-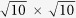 (Fig. 5b) with a better ordered surface than the Ca2-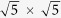, as seen in the Fourier transform pattern of the Ca2-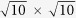, which is much sharper than the one of Ca2-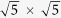 (see insets of Figs 3b and 4a). This suggests that an ordered surface favors superconductivity in this system. Thus, the absence of superconducting coherence peak in our STS spectra obtained from Ca-disordered Fe_2−x_Pt_x_As_2_ is due to the disorder of Ca atoms. However, this cannot explain why there is no coherence peaks seen in the bare Pt_4_As_8_ layer, where we observed an ordered structure (Fig. 5c). Here we propose that the lack of superconductivity on a bare Pt_4_As_8_ layer is a result of charge imbalance. In comparison with Ca2-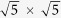 and Ca2-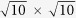 surfaces, the bare Pt_4_As_8_ layer has no Ca atoms, which must be responsible for the absence of superconducting coherence peaks. In the bulk, the charge transfer from Ca layer to Pt_4_As_8_ layer is much greater than the charge transfer from Ca to Fe_2−x_Pt_x_As_2_ layer^15^. The lack of Ca makes the Pt1-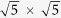 surface not superconducting which is much different than the bulk. Compared to the bare Pt1-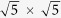 surface, the Ca2-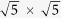 and Ca2-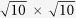 surfaces have much less severe charge imbalance, thus preserving superconductivity. The effect of charge imbalance is also evidenced at the surface of Sr_1−x_K_x_Fe_2_As_2_^27^, where no sign of superconducting coherence peak is observed on the ordered bare Fe_2_As_2_ layer. Moreover, it was shown that superconductivity can be tuned by charge transfer from gated ionic liquid to FeSe^28^.

In view of the previous studies of Fe-based superconductors including 11, 111, 122 systems, cleaving creates two identical surfaces, due to structure symmetry. A material like Ca_10_Pt_4_As_8_(Fe_2−x_Pt_x_As_2_)_5_ is fundamentally different offering great opportunities to study the structural and physical properties layer by layer, and provide important implication on the fabrication of new Fe-based superconductors. We have demonstrated that the electronic properties of the surface depend dramatically upon the stoichiometry and the arrangement of surface atoms. Cleaving and processing of the single crystal sample produce five distinct surface phases: a bare Pt_4_As_8_ surface (Pt1-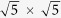), an ordered 0.2 ML Ca array on Pt_4_As_8_ surface (Ca2-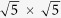), an ordered 0.1 ML Ca on Pt_4_As_8_ (Ca2-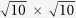), a Ca-clustered Pt_4_As_8_ layer, and a Ca-disordered layer on Fe_2−x_Pt_x_As_2_.

The fact that STS reveals no sign for superconductivity on the bare Pt_4_As_8_ surface and the disordered Ca on Fe_2−x_Pt_x_As_2_ indicates that both structure order and charge balance are crucial for superconductivity. We believe the unequal distance of Ca atoms with respect to both Fe_2−x_Pt_x_As_2_ layer and Pt_4_As_8_ layer is the reason for the existence of these strange surface structures. When the surface contains ingredients sufficient to maintain charge balance, properties seen in bulk can be preserved on the surface. This is supported by our observation of superconducting coherent peaks in STS on the Ca2-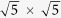 and Ca2-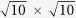 surfaces of Ca_10_Pt_4_As_8_(Fe_2−x_Pt_x_As_2_)_5_.

While three-dimensional superconductivity in layered superconductors is considered through tunneling between layers, our STM/STS study of Ca_10_Pt_4_As_8_(Fe_2−x_Pt_x_As_2_)_5_ reveals that proximity effect would not maintain the superconductivity on the surface if the surface experiences different chemical environment. As can be seen in Fig. 4e, both bare Pt_4_As_8_ layer and the disordered Ca layer are just about 3–4 Å from the Fe_2−x_Pt_x_As_2_ layer. The absence of superconductivity in these layers indicates that the top most Fe_2−x_Pt_x_As_2_ layer is not superconducting, even though the adjacent Fe_2−x_Pt_x_As_2_ layer is only ~8 Å below (Fig. 1a).

In summary, we have investigated the detailed surface structures and electronic properties of Ca_10_Pt_4_As_8_(Fe_2−x_Pt_x_As_2_)_5_ using STM/STS. We observe five different surfaces as summarized in Table 1. Remarkably, STS measurements reveal that only spectra taken on the Ca surfaces with *ordered structures* and *appropriate amount* show superconducting coherence peaks with finite zero bias conductance. Neither bare Pt_4_As_8_ surface nor disordered Ca/Fe_2−x_Pt_x_As_2_ exhibit superconductivity. Our results indicate that superconductivity can only be preserved on the surface when it has similar chemical arrangement as the bulk counterpart: either missing or disordered Ca would kill superconductivity in the surface planes, due to charge imbalance. Furthermore, our results confirm that the intermediate Pt_4_As_8_ layer is superconducting in Ca_10_Pt_4_As_8_(Fe_2−x_Pt_x_As_2_)_5_, direct evidence for bulk superconductivity.

## Methods

### Single crystal growth

High quality single crystals of Ca10-4-8 were grown using the self-flux method. The stoichiometric amounts of high purity calcium shot (99.999% Alfa Aesar), Pt powder (99.95% Alfa Aesar), iron powder (99.95% Alfa Aesar), and arsenic powder (99.999% Alfa Aesar) are mixed in the ratio 10:4:10:18. The mixture is placed in an alumina crucible and sealed in a quartz tube under vacuum. The whole assembly is heated in a box furnace to 700 °C at a rate of 150 °C/h and is held at this temperature for 5 h. It is further heated to 1100 °C at a rate of 80 °C/h where it is held for 50 h, and then cooled to 1050 °C at a rate of 1.25 °C/h. It is further cooled to 500 °C at a rate of 5.5 °C/h and finally cooled down to room temperature by turning off the power. Shiny black plate-like single crystals are obtained without requiring any additional process. These crystals have a typical size of 6 × 6 × 0.2 mm^3^.

### STM/S measurements

Ca10-4-8 single crystals are first cleaved at low temperatures (~90–100 K) then transferred to the scanning stage either at 80 K or 4.3 K. 10^10 ^V/A (current amplifier gain) is used at 4.3 K and 10^9^ V/A at 80 K so that different tunneling set currents are used at 4.3 K (2–40 pA) and 80 K (100 pA). No noticeable difference is observed in the surface topology taken at 80 K and 4.3 K. *dI/dV* spectra are acquired by using a lock-in amplifier with *V*_*mod*_ = 80 μV and *f*_*mod*_ = 555 Hz at 4.3 K. WSxM software was among the tools used for image preparation and analysis^29^.

### STEM measurements

STEM imaging is performed using a JEM-ARM200F microscope equipped with probe-corrector operating at 200 kV. Samples for TEM observation along different axis-zone direction are prepared by using Focused Ion Beam (FIB).

## Additional Information

**How to cite this article**: Kim, J. *et al*. Interrogating the superconductor Ca_10_(Pt_4_As_8_)(Fe_2−x_Pt_x_As_2_)_5_ Layer-by-layer. *Sci. Rep*. **6**, 35365; doi: 10.1038/srep35365 (2016).

## Supplementary Material

Supplementary Information

## Figures and Tables

**Table 1:** Summary of observed surfaces and their structures.

	Observed Surface	Referred Name	Lattice Size	SC
as-cleaved	bare Pt_4_As_8_ surface (no reconstruction): 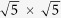 structure	“Pt1- 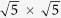 ”	8.7 Å: a square unit cell made of four Pt1 atoms	No
	reconstructed 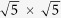 Ca surface on the Pt_4_As_8_ layer	“Ca2- 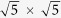 ”	8.7 Å: a square unit cell made of four Ca2 atoms	Yes
	disordered Ca surface on the Fe_2_As_2_ layer	“Ca-disordered”		No
after warming up to RT	Reconstructed 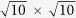 Ca surface on the Pt_4_As_8_ layer	“Ca2- 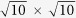 ”	12.3 Å: 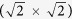 R45° reconstruction of “Ca2- 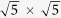 ”	Yes
after additional hour long annealing at RT	Ca clusters on the Pt_4_As_8_ layer	“Ca-clustered”		

The surfaces where superconductivity is observed are also marked.
