# Supplementary material for: Interrogating the superconductor Ca10(Pt4As8)(Fe2−xPtxAs2)5 Layer-by-layer
Source: Sci Rep. 2016 Oct 14;6:35365. doi: 10.1038/srep35365 (PMC5064410; doi:10.1038/srep35365)
Supplement: Supplementary Information [file srep35365-s1.pdf]

Supplementary Information: Interrogating the superconductor $Ca_{10}(Pt_4As_8)(Fe_{2-x}Pt_xAs_2)_5$ Layer-by-layer

Jisun Kim[1], Hyoungdo Nam[2], Guorong Li[1], A. B. Karki[1], Zhen Wang[1,3], Yimei Zhu[3], Chih-Kang Shih[2], Jiandi Zhang[1], Rongying Jin[1], and E. W. Plummer[1,*]

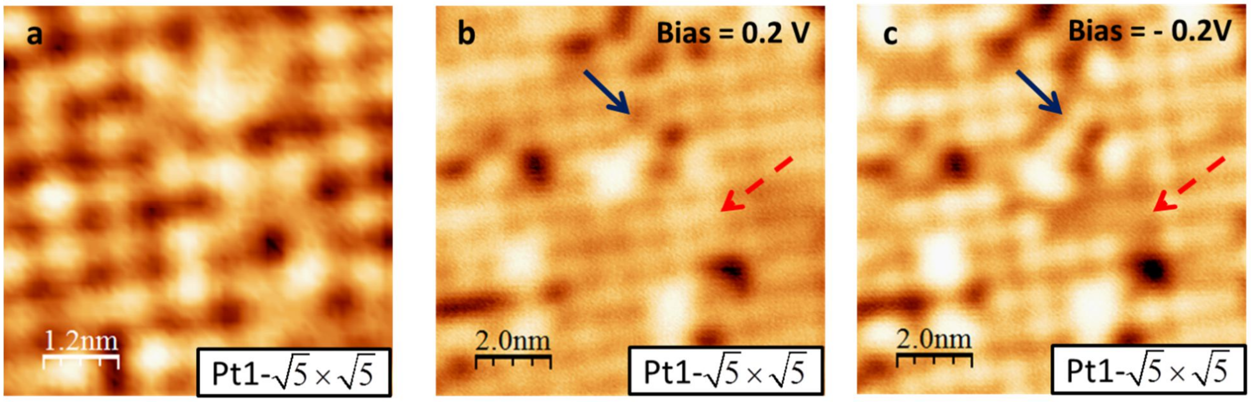

Supplementary Figure S1| **Local surface distortion of the Pt1-$\sqrt{5}\times\sqrt{5}$ surface:** (**a**) magnified image shows interconnection of certain Pt atoms which cannot be seen in larger images (V_{sample} = 20 mV, I = 40 pA), (**b – c**) bias dependent surface structure (I = 2 pA). Certain Pt atoms (marked with broken red arrow) seem to be missing when imaged at a negative bias. The electronic inhomogeneity may be related to Pt deficiency in the Pt_4As_8 layer reported previously.[12]

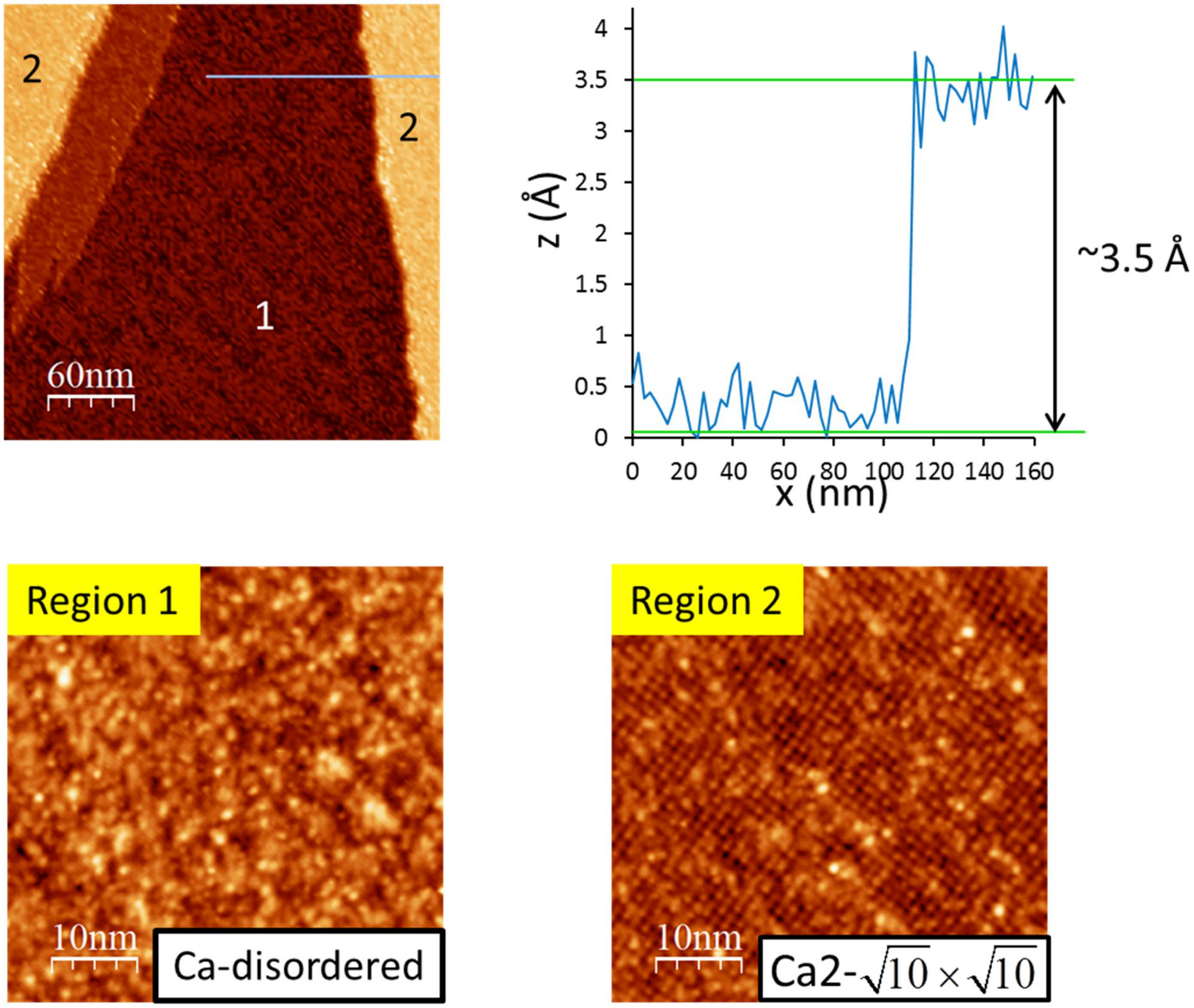

Supplementary Figure S2| **Ca-disordered surface and Ca2-$\sqrt{10}\times\sqrt{10}$ surface:** STM images of two different layers (the regions 1 and 2) after warmed from 4.3 K to room temperature (~ 290 K). The region 1 is disordered but the region 2 shows Ca2-$\sqrt{10}\times\sqrt{10}$ structure (the intermediate layer is due to the double-tip effect). The distance between the region 1 and 2 is about 3.5 Å, indicating that the region 1 is disordered Ca surface on the top of Fe_2As_2 layer. All images were acquired at V_{sample} = - 0.5 V and I = 2 pA.

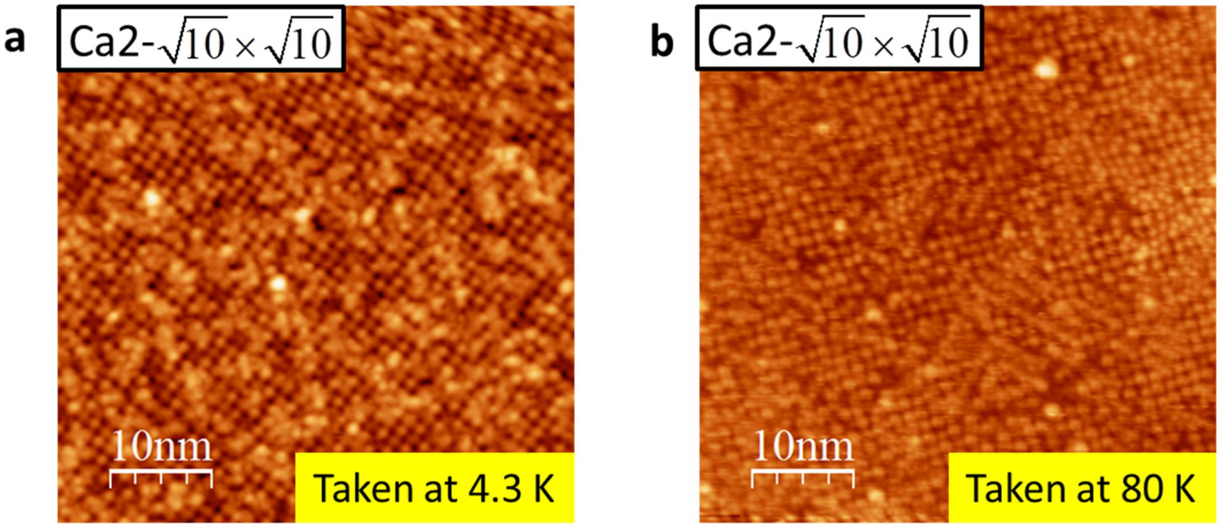

Supplementary Figure S3| **Ca2-$\sqrt{10}\times\sqrt{10}$ surface scanned at 4.3 K and 80 K:** STM image is taken at (a) 4.3 K, V_{sample} = - 0.5 V, and I = 2 pA; (b) 80 K, V_{sample} = 1 V, and I = 100 pA. There is no significant difference between (a) and (b).

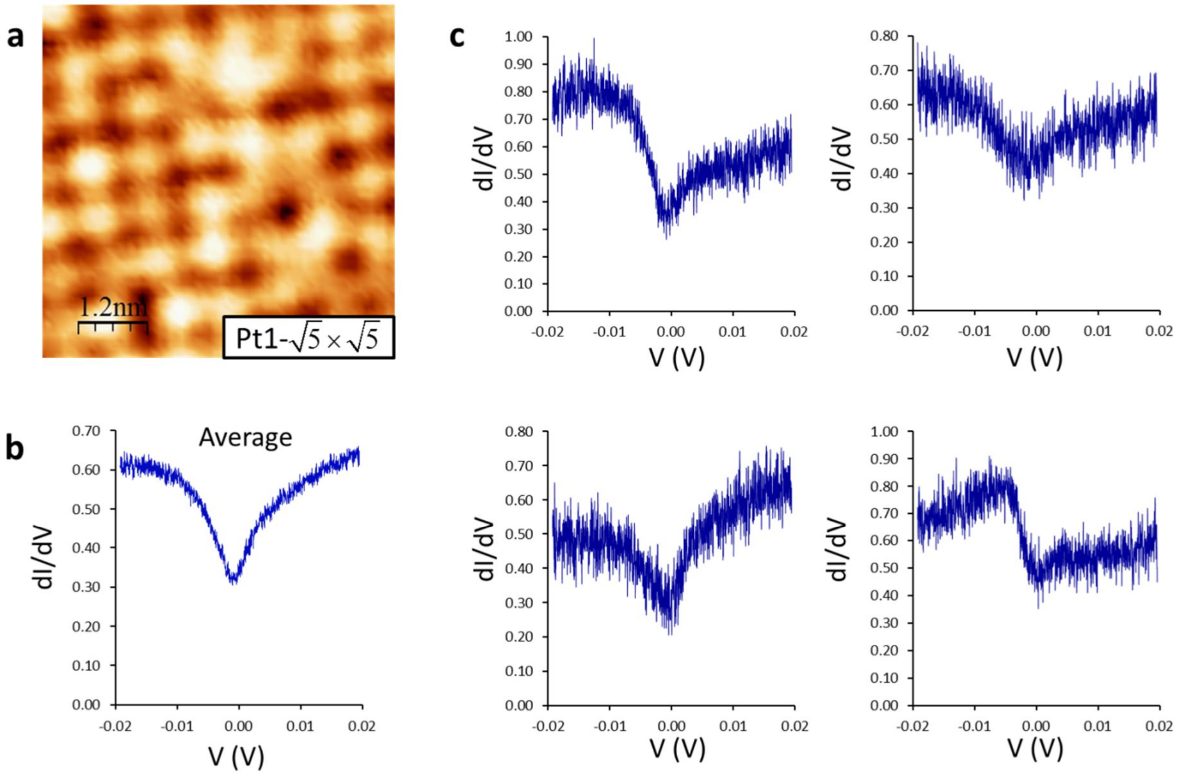

Supplementary Figure S4| **Site-dependent Pt1-** $\sqrt{5}\times\sqrt{5}$ **spectra:** (a) STM image of Pt1-$\sqrt{5}\times\sqrt{5}$ surface taken at 4.3 K (V_{sample} = 20 mV, I = 40 pA); (b) 16×16 spectra average taken on the shown surface; (c) individual spectrum at different locations in (a). The individual spectrum clearly shows that the detailed feature (e.g. shape and ZBC) varies significantly depending on the site where STS is taken, even though the surface is well ordered. None of site-dependent spectra shows coherence peaks, indicating the Pt1-$\sqrt{5}\times\sqrt{5}$ surface is not superconducting.
